# Reliability of Digital Artery Palpation and Pulse Oximetry Waveform As Alternatives to Radial Pulse in the Assessment of Upper Limb Injuries

**DOI:** 10.7759/cureus.68224

**Published:** 2024-08-30

**Authors:** Ahmed Elmahdi, Matthew Coombe-Jones, Edward Gee, Matthew Lea, Sammie-Jo Arnold, Amol Chitre

**Affiliations:** 1 Trauma and Orthopaedics, Salford Royal National Health Service (NHS) Foundation Trust, Manchester, GBR; 2 Trauma and Orthopaedics, Nottingham University Hospitals, Nottingham, GBR; 3 Trauma and Orthopaedics, Lancashire Teaching Hospitals Trust, Lancashire, GBR

**Keywords:** pulse oximetry waveform, alternatives, upper limb injuries, radial pulse, digital artery palpation

## Abstract

Introduction: Radial pulse palpation is widely accepted as a gold standard clinical method to assess distal vascular perfusion of the upper limb. In some instances, the radial pulse may not be accessible due to splints, casts, or swelling, or the injury may be at the level or distal to the radial artery. Here, the authors assess two alternative methods of assessing perfusion of the hand more distally: palpation of the digital pulse and pulse oximetry (PO) waveform.

Methods: Twenty-four healthy adult volunteers (48 hands) were assessed by two assessors. Digital artery pulses were palpated, and ease of location was recorded. A brachial cuff pressure was inflated to 20 mmHg above systolic pressure to occlude distal perfusion. Radial pulse, digital artery pulse, and PO waveform were monitored as the brachial cuff pressure was deflated in 5 mmHg increments to ascertain when each returned and compare the reliability of these tests to the gold standard of the radial pulse.

Results: The digital artery pulse was easily located in 20/24 participants, most reliably over the proximal phalanx of the index finger. With occlusion of the brachial artery, no distal pulses could be felt, and PO showed no waveform. As the brachial artery cuff pressure was incrementally deflated, the digital pulse returned with the same cuff pressure or a lower cuff pressure than the radial pulse in all cases, suggesting a high positive predictive value of radial pulse presence. PO waveform returned at a higher cuff pressure or with the same cuff pressure as the return of the radial pulse, suggesting a higher sensitivity than radial pulse palpation in assessing hand perfusion.

Conclusion: Digital pulse palpation can be used as a surrogate method of assessing hand perfusion. When present, it can be assumed a radial pulse is present due to a high positive predictive value and no false positives seen in any participant. When absent, further investigation is required. The PO waveform was found to be more sensitive than digital or radial pulse palpation as a measure of distal perfusion, with a return of waveform prior to palpable pulses. This likely represents a more accurate clinical test of distal perfusion and can be relied upon even when pulses are not palpable.

## Introduction

In limb injuries, distal perfusion of the limb is a key aspect of initial assessment and contributes to the basis for management decisions [1-4]. Documentation of radial pulse palpability is currently the most widely accepted standard in clinical assessment of distal perfusion of the upper limb and forms part of trauma guidelines such as the British Orthopaedic Association Standards for Trauma [5,6].

It is often the case that acute care providers have applied an immobilizing plaster cast or other device such as a splint or sling to an injured upper limb before orthopedic assessment. Any immobilizing device is likely to obscure the wrist, preventing examination of the radial pulse without opening or removing the immobilization. This is time-consuming, may cause pain or distress to the patient, and will weaken the stability of a plaster cast [7]. It may also be the case that swelling occurs over the radial pulse, inhibiting examination, or that the injury occurs at the level of the wrist crease or distal, requiring a more distal method of assessment that is more reliable than capillary refill [8].

In this two-part study, we examine two alternative methods of assessing perfusion of the hand. Firstly, the ease of identification of digital artery pulse was assessed, and the most reliable anatomical points for palpation were recorded. The assessors had pre-existing knowledge of the anatomical course of the digital arteries [9]. Digital pulse palpation and the presence of a waveform on pulse oximetry (PO) were then compared to the accepted standard of radial pulse palpation as indicators of distal perfusion. Comparison consisted of comparing the points at which pulses and waveforms returned as a brachial cuff pressure was deflated and perfusion to the hand was restored.

## Materials and methods

A search of the English, published literature was performed using Pubmed, Google Scholar, and the Cochrane Library on three separate dates in May 2022 by two authors. The search terms were "digital pulse" OR “digital artery” OR “pulse oximetry” AND “radial pulse" OR “radial artery” OR “perfusion” with no other limits. Results were reviewed and the references of appropriate studies were inspected for further relevant articles. This revealed no articles discussing digital artery pulse palpation as a method of assessing distal upper limb perfusion or comparing it to the radial pulse or PO.

Twenty-four healthy adult volunteers were examined by two assessors (EG and ML). The volunteers were made up exclusively of fourth-year medical students, with an age range of 21 to 24 years old and an even ratio of male to female. The temperature of the room was kept at a constant 22˚ Celsius, and all participants were given a minimum of 15 minutes of rest and a glass of room temperature water once arriving at the examination location for blood pressure and heart rate to return to baseline. Assessors were blinded (facing in opposite directions), and examinations were performed without conference. The assessors could, therefore, not see the cuff pressure readings to avoid bias and were not aware of each other's recorded results on each patient. Based on the results of a pilot study, set anatomical points were identified as common areas where the digital pulse may be easily palpable. These set points consisted of either side of the flexor tendons at the bases of the fingers and the midpoints of both the proximal and middle phalanges. The thumb was excluded (as the base of the thumb is often concealed in plaster casts, backslabs, and wrist splints).

Exclusion of the thumb also allowed PO assessment on the thumb throughout, without proximal digital artery palpation on the same digit, which could have affected PO readings. A PO waveform was observed on the digital monitor (Mindray VS-900, Mindray (UK) Limited, United Kingdom).

Firstly, the two assessors palpated each pre-set anatomical point independently. The palpated pulse was matched to the PO waveform to ensure that the pulse was that of the volunteer and not the assessor. The assessors recorded each set point on a proforma as “not palpable,” “palpable,” or “easily palpable.” The assessors had a maximum of 10 seconds at each point to identify the pulse to ensure it could be located with ease and speed. Both hands were examined separately by each assessor. Proformas were collated by a third assessor (SA) to ascertain the most easily available digital pulse in each hand.

A manual sphygmomanometer was used to establish the systolic blood pressure midway along the arm over the brachial artery. The cuff was then inflated to 20 mmHg above the systolic pressure to ensure that the brachial artery was completely occluded, with the absence of pulses and PO waveform.

The pressure in the cuff was then manually reduced in 5 mmHg increments by the third assessor (SA), pausing for five seconds at each increment. Pulse points were palpated gently (to reduce compression), and a digital monitor was observed for PO waveform by the other two assessors (EG, ML).

Once the radial pulse returned, palpation at this level stopped, and the digital pulse and pulse oximetry were examined for a further five seconds to ensure they had not been affected by more proximal palpation. A record was made of the cuff pressure that each pulse and the PO waveform returned in relation to the participant’s systolic pressure.

## Results

Twenty-four volunteers (48 hands) were assessed, with an equal spread of sex and an average age of 22 years (21-25). Digital artery pulsation was easily palpable in 20 participants (83%); in the other four participants, the digital pulse could not be reliably felt by either assessor, giving a false negative rate of 4/24 (16.7%). These four participants (eight hands) were excluded from the rest of the study. In no individual was the digital pulse present without an easily palpable radial pulse (false positive rate of 0/24).

The most easily palpable digital pulse in each hand was recorded, most commonly on the ulnar side of the proximal phalanx on the index (15/40 hands) and middle fingers (10/40 hands) (Table 1).

**Table 1:** Location of the most reliable pulse on 40 hands (20 participants)

Finger	Side of finger	Proximal phalanx	Middle phalanx
Index	Radial	0	0
	Ulnar	15	1
Middle	Radial	1	1
	Ulnar	10	3
Ring	Radial	3	3
	Ulnar	2	1

The mean systolic blood pressure (taken manually) at the brachial artery in seated individuals was 123 mmHg. When the brachial artery was occluded, all 40 hands had no palpable radial artery pulse, digital artery pulse, or PO waveform. As the cuff pressure was deflated, the radial pulse returned with the cuff pressure ranging from 5 to 30 mmHg below the systolic pressure (mean 16 mmHg below systolic, absolute pressure 107 mmHg).

The digital pulse returned with a mean cuff pressure that was 29 mmHg below the systolic blood pressure and 13 mmHg below the pressure at which the radial pulse returned. Digital pulse returned at the same (n=2/20) or lower cuff pressure (n=18/20) than radial pulse return in all cases, with a maximum difference of 25 mmHg (n=4/20) (Table 2.) There were no false positives of a palpable digital pulse without a radial pulse in any participant. In all cases, the PO waveform returned at the same (n=10) or at a higher (n=10) cuff pressure than the return of the radial pulse, with a maximum difference of 10 mmHg (n=5) (Table 2).

**Table 2:** Comparisons of the brachial cuff pressures at the return of radial pulse to cuff pressures at the return of digital pulse and waveform on PO

Cuff pressure difference (mmHg)	Digital pulse return vs. radial pulse return	“Pulse wave” return vs. radial pulse return
10	0	5
5	0	5
0	2	10
-5	2	-
-10	7	-
-15	0	-
-20	5	-
-25	4	-

## Discussion

In upper limb trauma, documentation of the radial pulse is widely accepted as an indicator of more proximal artery patency and pertains to distal limb perfusion [5]. In certain circumstances (such as a cast covering the wrist), this pulse may not be accessible or reliable, and a more distal alternative may be sought. Capillary refill is often relied upon in these instances but is clinically limited in a vasoconstricted limb or a limb with pigmented skin and can give false reassurance with venous blood returning to the capillaries rather than arterial perfusion [10].

This study’s main aim was to ascertain whether the palpation of the digital artery pulsation or the presence of a waveform on PO trace could be used as reliable alternatives to radial artery palpation in the assessment of hand perfusion in a patient with upper limb trauma.

In the literature search, no equivalent studies were identified. However, two studies were identified that explored the use of PO both for assessing distal perfusion of the digits in the presence of vascular injuries of the hand [11] and the use of PO for assessing distal perfusion in the presence of brachial artery injury [12]. Both studies concluded that PO was an effective technique for assessing distal perfusion and the presence of an arterial pulse.

Our study found that in the majority of healthy adult individuals (20/24) the digital pulse is easily palpable, and the most reliable location was on the ulnar side of the proximal phalanx, of the index (15/40 hands) or middle fingers (10/40 hands). The little finger rarely had a palpable digital pulse, and the thumb was excluded. In four individuals, the digital pulse could not be reliably felt in any of the digits (participants excluded). One of these individuals had a diagnosis of Raynaud’s phenomenon; they were peripherally vasoconstricted with a "weak" radial pulse. There were no known medical causes for the other three participants, and it may be a normal anatomical variant. All volunteers with a palpable digital pulse had a reliable radial pulse.

**Figure 1:**
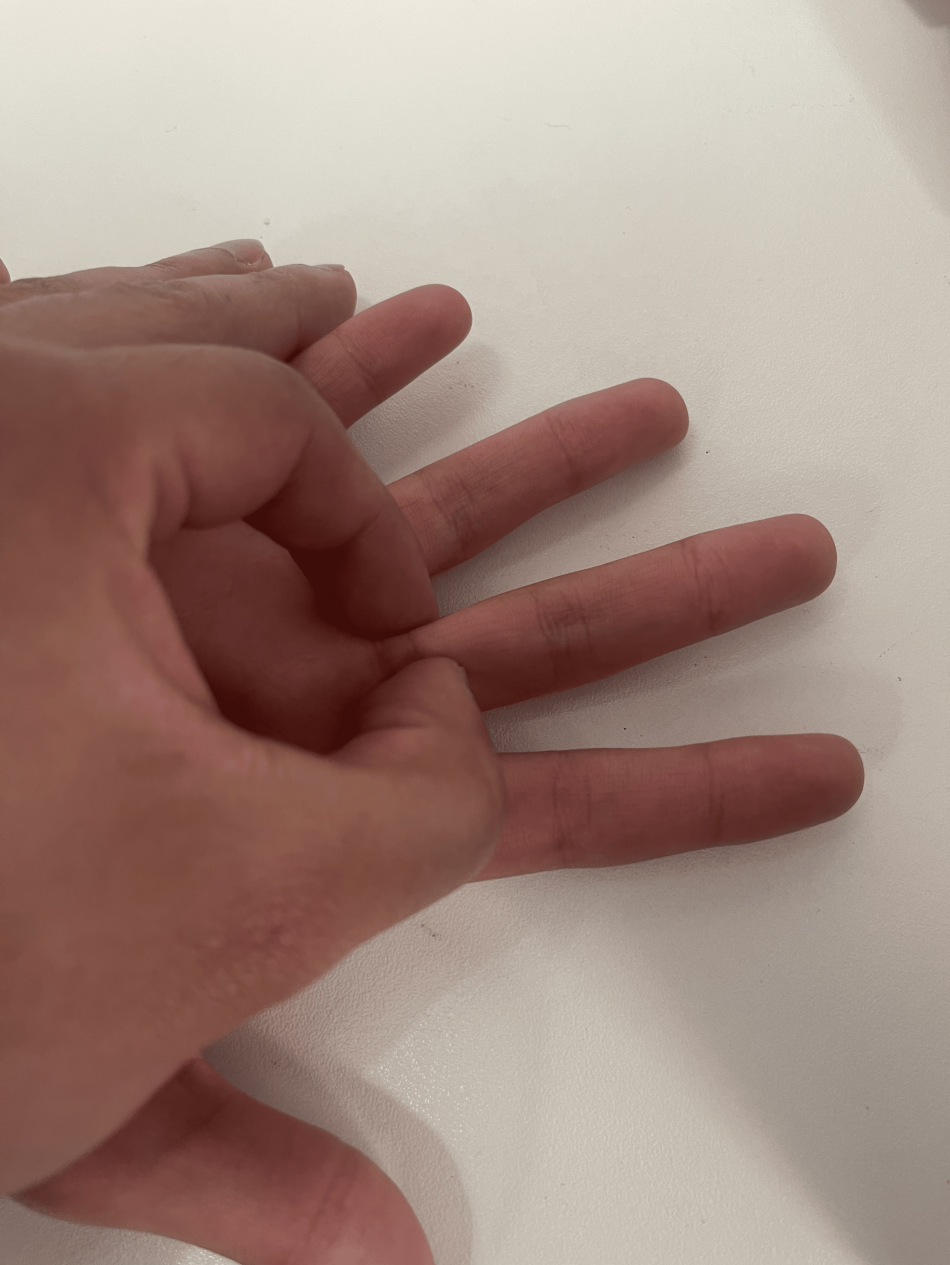
Palpation technique

The most reliable method for palpating digital pulse was using a gentle pincer grip of the examiner's index finger and thumb, either side of the flexor tendons on the volar aspect of the index and middle fingers (Figure 1).

As the cuff pressure was incrementally deflated, palpation of the digital and radial arterial pulses simultaneously enabled a direct comparison of when the pulses became palpable. Radial pulse was palpated gently to avoid compression and limitation of distal flow. Once the radial pulse had returned, palpation at this level was removed immediately to remove any residual compression, and the digital pulse was re-examined.

The actual pressure value on the cuff is insignificant, but its relationship to systolic pressure and the comparison between pressures at which each pulse and the PO waveform signal returned do have significance. If the digital pulse and PO waveform returned at the same cuff pressure point as the radial pulse, they could be considered as useful and as sensitive as the currently accepted clinical method. If digital pulse and PO waveform returned after radial pulse, then the clinical application would be that their presence suggests hand perfusion as reliably as a radial pulse and their absence justifies further examination. If the digital pulse and PO waveform returned at a higher cuff pressure than the radial artery, then they could be present without perfusion being robust enough to provide a palpable radial pulse. This could be interpreted as a false positive; however, in reality, this would suggest a more sensitive test of distal perfusion than the radial pulse. If their relationship to radial pulse is inconsistent, then they are unreliable as an alternative to radial pulse and have no clinical use.

In our study, we demonstrated that although the digital pulse was not palpable in every volunteer (4 out of 24 did not have a reliably palpable pulse), the digital pulse was never present without a palpable radial pulse and consistently (100% of included participants) returned with the brachial cuff pressure the same (n=2) or lower (n=18) than the pressure at which the radial pulse returned, with a range of cuff pressures from 0 mmHg to 25 mmHg below that of the systolic pressure.

The presence of a palpable digital artery pulse has statistical significance as a positive predictor (100%) of radial pulse presence with compression at the level of the brachial artery. The absence of the digital pulse, however, has less statistical relevance (85.7%) and does not directly correlate to a lack of a palpable radial pulse. The absence of a palpable digital artery pulse, however, does justify the inconvenience and discomfort of further examination that may involve removing or partly splitting immobilizing splints or casts or applying other methods to confirm the status of distal limb perfusion.

The waveform returned to the PO monitor when the brachial cuff pressure was within 10 mmHg above the pressure at which the radial pulse returned in all individuals. As the brachial cuff pressure was reduced, the PO waveform returned prior to a palpable radial pulse in 50% (10 out of 20) of patients and at the same brachial cuff pressure as the return of the radial pulse in the other 50% (10 out of 20) of patients. This suggests that although the PO waveform pattern may be present without a palpable radial pulse, this may be considered a more sensitive test for capillary perfusion of the hand than clinical palpation of the radial artery and a useful second-line test if perfusion is uncertain.

According to these results, in a proximal injury at the level of the brachial artery, if digital pulses can be felt, then a radial pulse can be assumed to be present unless direct injury or compression of the radial artery has occurred simultaneously. If the digital pulse is not palpable, then further investigation is warranted with PO or opening a cast to examine the radial pulse. PO waveform can be present without a palpable digital or radial pulse but can be considered a reliable, more sensitive indicator of distal capillary perfusion and confirms the patency of proximal vessels.

Due to the anastomotic supply of the arterial arcade of the hand [9], however, these clinical tests could be positive despite occlusion or injury to the radial artery. For this reason, it is important to be clear that these tests do not directly confer radial artery patency or radial pulse presence. More realistically, they provide alternative methods of assessing perfusion of the digits. For this reason, in distal injury, these tests should be performed on multiple digits.

Despite the efforts of the authors to control blood pressure dynamics (constant temperature, glass of water, and 15 minutes of relaxation prior to study commencement), the authors accept the limitations of a blood pressure study and that results may have been altered by changes in systolic blood pressure due to pain response secondary to prolonged cuff inflation time [13] or alterations in pressures after restoration of perfusion and return of ischemic factors on cuff deflation [14].

Other limitations to acknowledge include our relatively small sample size of 24 and our sample population of generally young and healthy individuals. Results may vary in an older, more comorbid sample population.

## Conclusions

In a patient with upper limb trauma at the level of the brachial artery, where the radial pulse is not accessible, an alternative method of assessing distal perfusion may be beneficial to avoid the unnecessary discomfort and distress of removing immobilization. The palpation of a digital artery pulse is a reliable indicator of a present radial pulse in the uninjured patient, with no false positives. In injured patients, it would be a reliable indicator of distal perfusion. The digital pulse is usually best felt on the ulnar aspect of the volar surface of the index and middle finger proximal phalanges. If the digital pulse cannot be palpated, further examination is justified with pulse oximetry assessment or partial removal of an obscuring cast, splint, or dressing to examine the radial pulse. The use of a PO probe and display to look for a waveform potentially provides a more sensitive indicator of distal capillary perfusion, as a waveform can be present with a lower brachial arterial flow than is required to provide a palpable radial pulse.
